# Comparison of non-viral methods to genetically modify and enrich populations of primary human corneal endothelial cells

**Published:** 2009-04-01

**Authors:** Christoph Engler, Clare Kelliher, Karl J. Wahlin, Caroline L. Speck, Albert S. Jun

**Affiliations:** Wilmer Eye Institute, The Johns Hopkins Medical Institutions, Baltimore, MD

## Abstract

**Purpose:**

To compare different techniques of transfection of primary human corneal endothelial cells (HCECs) by non-viral methods and to enrich genetically modified cells to a highly pure population.

**Methods:**

HCECs were cultured following previously published methods. Dissection of the Descemet membrane (DM) was performed by tearing off strips from corneal buttons with forceps or by hydrodissection. Confirmation of HCECs identity was performed by reverse transcriptase polymerase chain reaction (RT–PCR) for α2 collagen VIII. For transfection, non-viral methods such as lipid-/liposome-mediated reagents and electroporation techniques were compared. Genetically modified cells were enriched by use of selection antibiotics and flow cytometry.

**Results:**

Viability of primary HCECs was lower in hydrodissected corneas. The rate of transfection varied from approximately 5%–30%. Highest rates of transfection were obtained with the Amaxa electroporation method. The next highest rate was yielded by the lipid-mediated reagent GenCarrier2, followed by electroporation with the BTX apparatus. Toxicity was moderate and manageable by adjusting the concentration of reagents, incubation times, and electrical parameters. Selection by flow cytometry was superior to antibiotic selection and produced nearly 100% genetically modified cells.

**Conclusions:**

Electroporation of HCECs yields higher transfection efficiency than chemically mediated methods. It is possible to select genetically modified HCECs to high levels of homogeneity. Techniques to genetically modify and select HCECs as shown in this study could lead to improved success of future endothelial transplant procedures.

## Introduction

Corneal endothelial failure is a major indication for corneal transplantation. A leading cause of endothelial failure is Fuchs endothelial dystrophy (FED). This disease is characterized by a slow, continuous loss of morphologically and physiologically altered endothelial cells, eventually leading to corneal edema and a thickened Descemet membrane (DM) with focal excrescences of altered basement membrane material (*guttae*) [1,2]. Collagen VIII, which includes α1 and α2 chains, is produced by corneal endothelial cells and is an important structural component of DM. At least two different mutations in the α2 chain of collagen VIII (*COL8A2*) have been shown to cause early-onset FED [3,4]. Finding a way to use modified human corneal endothelial cells (HCECs) as an approach for cellular or gene therapy and to avoid the need for corneal transplantation would be a promising goal. The objective would be to either restore the physiologic functions of diseased endothelial cells or to successfully transplant healthy endothelial cells alone.

Potential therapeutic uses of cultured HCECs may improve from the ability to genetically modify these cells in a safe and reliable manner. To date, the highest rates of genetic modification of HCECs have been reported for viral vectors, and less promising results have been reported for genetic modification of HCECs by non-viral methods. The use of viral vectors bears potential risks such as malignant transformation and immune responses to viral proteins [5]. In addition, the ability to select genetically modified HCECs to a high level of homogeneity may yield advantages if such cells were to be used for a therapeutic application. In this study, we directly compared non-viral methods of transfection such as lipid-/liposome-mediated reagents and electroporation techniques for transfection efficiency and toxicity in HCECs. We also investigated antibiotic selection and flow cytometry as methods to enrich populations of genetically modified HCECs.

## Methods

Research methods described adhered to the tenets of the Declaration of Helsinki.

Serum-free medium (OptiMEM-I, Dulbecco), phosphate-buffered saline (PBS), and gentamicin and trypsin- ethylene diamine tetraacetic acid (EDTA) were obtained from Gibco BRL/Life Technologies (Rockville, MD). Mouse submaxillary gland nerve growth factor and bovine pituitary extract were obtained from Biomedical Technologies (Stoughton, MA), and mouse submaxillary gland epidermal growth factor was obtained from Upstate Biotechnologies (Lake Placid, NY). Fetal bovine serum (FBS) was obtained from Hyclone (Logan, UT), and ascorbic acid, chondroitin sulfate, calcium chloride, 0.02% EDTA solution, dimethylsulfoxide (DMSO), antibiotic antimycotic solution, agarose, ethidium bromide, and 0.4% trypan blue were obtained from Sigma (St. Louis, MO). The following materials were obtained from companies noted in parentheses: cell attachment reagent (FNC coating mix; Athena Environmental Sciences Inc., Baltimore, MD), Fugene (Roche Diagnostics, Basel, Switzerland), lipofectin, Plus Reagent, TAE buffer, DNase I Amplification Grade (all from Invitrogen, Carlsbad, CA), GenCarrier2 (Epoch Biolabs, Missouri City, TX), plasmids pIRES-EGFP and pEGFP-N1 (BD Biosciences Clontech, Mountain View, CA), and Amaxa Reagent Solution Kit for mammalian endothelial cells, pmaxGFP (Amaxa Inc., Gaithersburg, MD).

### Human corneal endothelial cell culture

Corneas from donors aged 27−73 years stored in corneal preservation media (Optisol-GS; Bausch & Lomb, Rochester, NY) were obtained from Tissue Banks International (Baltimore, MD).

Human endothelial cell lines were established according to previously published protocols [6,7]. Corneas were removed from corneal preservation media and were washed with culture medium consisting of Opti-MEM I, 8% FBS, 5 ng/ml epidermal growth factor (EGF), 20 ng/ml nerve growth factor (NGF), 100 µg/ml pituitary extract, 20 µg/ml ascorbic acid, 200 mg/l calcium chloride, 0.08% chondroitin sulfate, 50 µg/ml gentamicin, and antibiotic antimycotic solution diluted 1/100.

Two methods were used to remove DM with intact endothelium. In the first method, strips of DM and endothelium were dissected from corneal buttons. In a second method, sterile saline was carefully injected in the posterior stroma with a fine needle. This leads to the formation of a bleb under DM. After expansion of this bleb almost to the limbus, the saline was carefully removed. DM together with endothelial cells was incised with a trephine blade and removed from the underlying stroma.

The harvested DM/endothelial tissues were then incubated in culture medium overnight at 37 °C under 5% CO_2_ and were then centrifuged at 504x g for 6 min and incubated in 0.02% EDTA for 1 h at 37 °C to loosen cell-cell junctions. The tissue was then manually disrupted by passage through a flame-polished glass pipette. The dissociated cells were centrifuged again at 504x g for 6 min, and the pellet was gently resuspended in culture medium.

In another method, the DM and endothelial cell strips were incubated for 30–60 min in trypsin-EDTA to enhance the loosening of the cell-cell junctions before passage through a flame-polished pipette.

Isolated cells and pieces of DM were plated in a single well of a six-well tissue culture plate pre-coated with an undiluted cell attachment reagent. Cultures were incubated at 37 °C in a 5% CO_2_ humidified incubator, and the medium was changed every other day. Once the cells approached approximately 80%–90% confluence, the cells were treated with trypsin-EDTA and passaged at suitable ratios. Cryopreservation was performed as described previously [8]. Cell lines used for transfection experiments had been passaged between four and six times, and some cell lines had been used thawed after cryopreservation.

### Human corneal stromal fibroblast culture

Stromal fibroblast cultures were established as follows. The epithelium and endothelium were removed from the corneal buttons. The corneal button was left in culture medium (same as that used for growing endothelial cells) for one to two weeks until a monolayer of spindle-shaped cells (stromal fibroblasts) was established along the bottom of the tissue culture plate. The medium was changed, and cells were passaged at appropriate time intervals.

### Reverse transcriptase polymerase chain reaction

Total RNA was prepared for reverse transcriptase polymerase chain reaction (RT–PCR) from cultured HCECs, which were 70–80% confluent at the time of harvest. A commercially available kit was used (RNeasy Mini Kit; Qiagen, Valencia, CA).

RT–PCR for *COL8A2* was performed on a thermal cycler (DNA engine; Bio-Rad, Hercules, CA). Primers for *COL8A2* spanned one intron to distinguish amplification of genomic DNA. Primer sequences were as follows: sense primer: 5′-ATC CAG CCC ATG CAG AAA G-3′, antisense primer: 5′-GCT CTC CCT TCA GGT CCA T-3′. The expected product length was 106 base pairs. For *β-actin*, a commercially available primer set was used (Stratagene, La Jolla, CA). Sequence of the sense primer was 5′-TGA CGG GGT CAC CCA CAC TGT GCC CAT CTA-3′. The antisense primer sequence was 5′-CTA GAA GCA TTT GCG GTG GAC GAT GGA GGG-3′. As a negative control, total RNA of human mononuclear cells (StemCell Technologies Inc., Vancouver, Canada) was used.

RT–PCR was performed for 25 cycles using commercially available reagents and protocols (SuperScript One-Step RT-PCR System with Platinum Taq DNA Polymerase; Invitrogen). RT–PCR steps were as follows: 1 cycle of 30 min at 55 °C and 2 min at 94 °C followed by 25 cycles of 15 s at 95 °C, 1 min at 50 °C, 1 min at 72 °C, and 1 cycle of 5 min at 72 °C. DNase treatment of RNA samples was performed to rule out genomic DNA contamination following manufacturer’s protocols (Roche Applied Diagnostics, Mannheim, Germany). Samples were electrophoresed in 2% agarose gels containing ethidium bromide and photographed using standard methods.

### Transfection of human endothelial cells

#### Lipid-/Liposome-mediated methods

For transfection, the following commercially available lipid-/liposome-mediated reagents were used and optimized according to manufacturer’s protocols: Fugene, lipofectin, Plus Reagent, and GenCarrier2. Transfection procedures were performed on attached cells with Fugene and lipofectin. With Fugene, various ratios of reagent (µl):DNA (µg) were compared (3:1, 3:2, 6:1, 9:1, and 12:1). With lipofectin, reagent:DNA ratios tested were 5:1, 10:1, 15:2, 20:2, and 25:2. PlusReagent was added according to manufacturer’s recommendations in some experiments for enhancement of transfection efficiency. Incubation time was 18 h or 24 h. With GenCarrier2, transfection was performed on cell suspensions. Ratio of reagent:DNA was 18:3, and incubation time was 45 min. Confluence of cells on the day of transfection was 50%–70%. The cell line used was from a 38-year-old donor, harvested by forceps stripping. Cells had been thawed after cryopreservation. Each condition was performed at least three times.

We used plasmids, pIRES-EGFP and pEGFP-N1, both of which contain an enhanced green fluorescent protein (EGFP) marker gene as well as a kanamycin/neomycin resistance gene. The plasmid, pmaxGFP, without a neomycin resistance gene was used with the Amaxa method (see next paragraph). Transfection procedures were optimized using manufacturer’s protocols. The rate of transfection was monitored by fluorescence microscopy. In some cases, a selection antibiotic (neomycin) was added (200–300 µg/ml) 48 h after transfection.

#### Electroporation

Two different electroporation methods were compared, BTX (BTX electroporator ECM 830, BTX Instrument Division; Harvard Apparatus, Holliston, MA) and the Amaxa Nucleofector II (Amaxa). BTX electroporation was performed with various electric parameters. The first setting consisted of three pulses, pulse length 1 ms, interval 100 ms. Voltage ranged from 50 to 200 V. In a second setting we applied one pulse, pulse length 70 ms. Voltage applied also ranged from 50 to 200 V. In all experiments, a cuvette with a 2 mm gap was used. Cell line used was the same as the one used with lipid-/liposome-mediated methods (38-year-old donor, harvested by forceps stripping, cells had been thawed after cryopreservation). Each setting was performed once.

With the Amaxa electroporation method, we used the specific reagents and programs recommended for mammalian endothelial cells (programs M-03, T-05, T-23, T-27, and U-11). All five programs were tested once. In the following four replicates, only programs T-23 and T-27 were used. Cell lines used were from donors aged 24, 38 (in two out of five experiments), 27, and 66 years. All cell lines were harvested by forceps stripping except for one case and had been cryopreserved before use.

### Selection by antibiotics/flow cytometry with fluorescence-activated cell sorting

In experiments with neomycin, the minimum dose required to kill primary, non-transformed cells had been determined previously to be 300 µg/ml. Neomycin was added to the growth medium 48 h after transfection. In all cases, neomycin was removed from the medium after 10 days. Flow cytometry with fluorescence-activated cell sorting (FACS, MoFlo High-Performance Cell Sorter; Cytomation, Fort Collins, CO) for EGFP expression was performed to select fluorescent cells into a highly pure population.

### Light and fluorescence microscopy

Cells were viewed and photographed with an inverted light and fluorescent digital microscopy/photography system (Axiovert 2000 M; Carl Zeiss Microimaging, Thornwood, NY).

### Statistical analysis

Determination of transfection rate was performed by counting EGFP-positive cells using fluorescence microscopy. Statistical analysis was performed with Microsoft Office Excel 2003 (Microsoft, Redmond, WA) and GraphPad Prism 4 (GraphPad, La Jolla, CA). p values were calculated using an unpaired *t*-test or χ^2^ test. p values less than or equal to 0.05 were considered statistically significant.

## Results

### Culturing of human corneal endothelial cells

**Figure 1:**
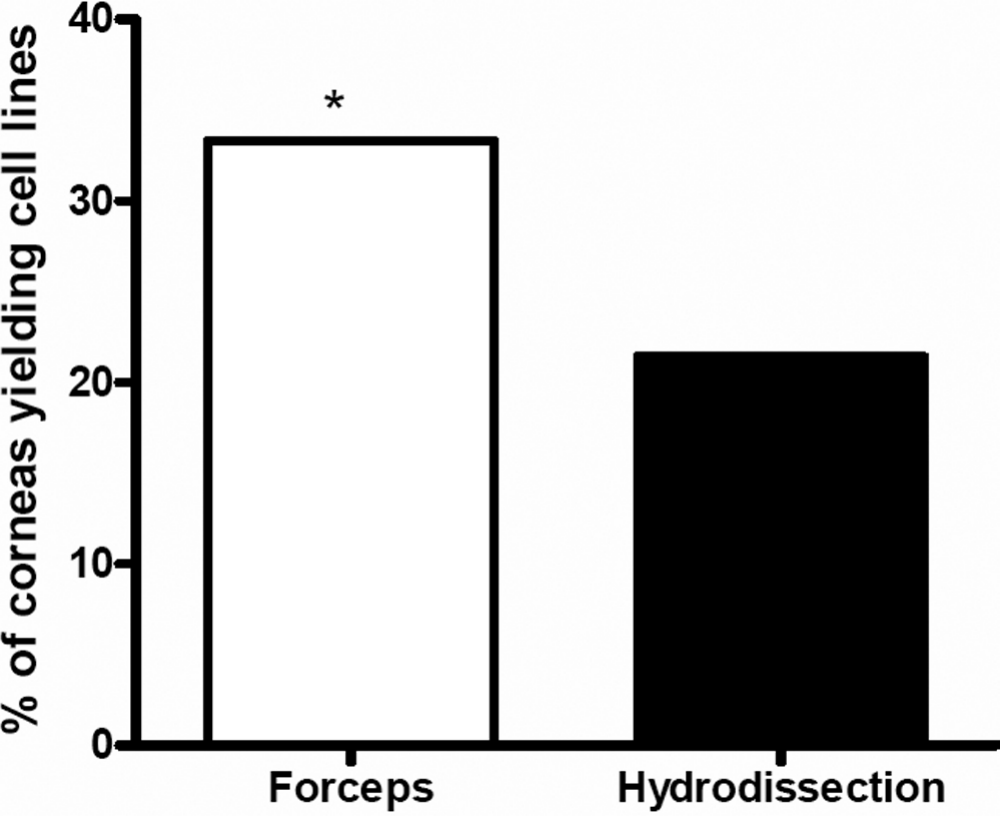
Comparison of success of harvesting methods. Forceps stripping yielded successful cell lines in 33.3%, and hydrodissection in 21.4% of all corneas used with these methods, respectively. The asterisk indicates p≤0.05 (χ^2^-test)

**Figure 2:**
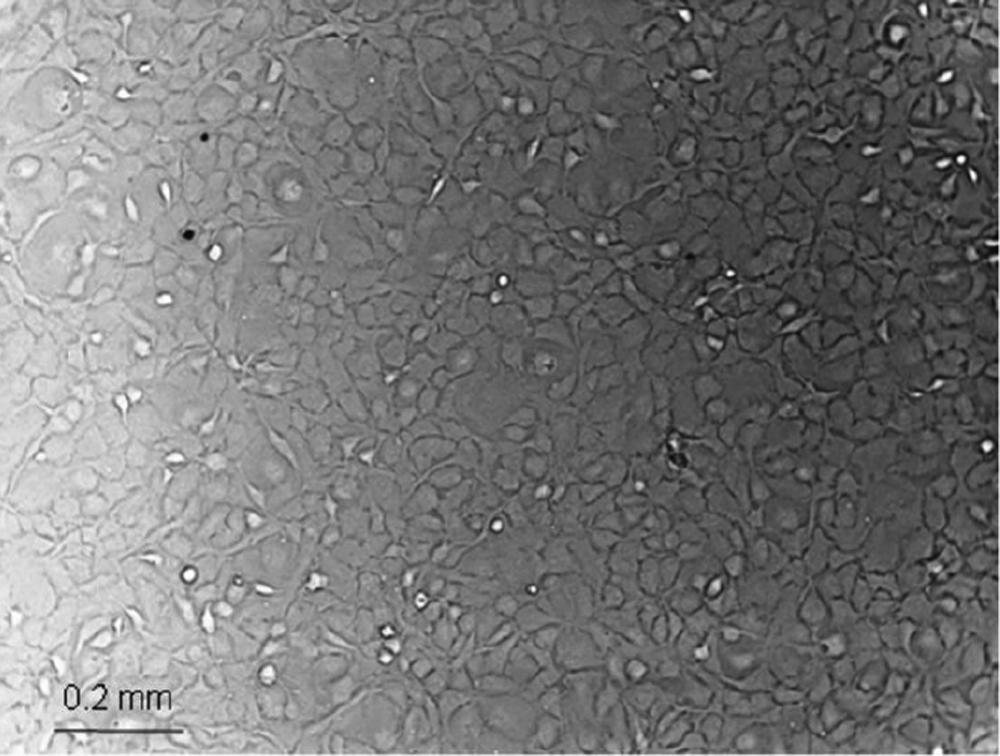
Human corneal endothelial cells after 10 days of culturing before first passage. Cells exhibited a polygonal shape similar as in vivo.

Thirty-five corneas were obtained to attempt culturing of HCECs (Table 1). The donor age was 53±16 (mean±SD) years. Cell lines were established successfully for 10 (29%) of the corneas. Age (mean±SD) of the donors yielding cell lines was 50±19 years, and age of donors with failed cultures was 54±15 years (p>0.05). Days in storage of successful corneas were 9±6. Days in storage of failed corneas were 9±4 (p>0.05). Endothelial cells of older donors tended to grow more slowly and often stopped growing after one to two weeks without reaching confluence. These cells were more often irregularly formed and more often exhibited a fibroblastic phenotype as reported previously [9]. Hydrodissection was performed in 14 corneas. Hydrodissected corneas resulted in fewer viable cell lines compared to the ones stripped with forceps. Hydrodissection yielded viable cell lines for 3 out of 14 (21.4%) corneas whereas forceps stripping was successful for 7 out of 21 (33.3%) corneas. Performing χ^2^ test to compare success rates of the two different harvesting techniques showed a significantly higher success rate with forceps stripping over hydrodissection (p≤0.05; Figure 1). Age of the donors used for hydrodissection was 55±16 (mean±SD), and age of donors used for stripping was 52±17 years (p>0.05). Corneas stained with 0.4% trypan blue for 1 min before removing the Descemet membrane showed complete endothelial cell death several days after harvesting. In successfully cultured cells, the ability to proliferate was maintained for several months. Before reaching confluence, cells had an uneven, sometimes stretched shape, which resembled to some extent a fibroblastic appearance. After reaching confluence, cells exhibited a rounder, polygonal shape more similar to the morphology of endothelial cells in vivo (Figure 2). This appearance was especially obvious before the first passage. Cryopreservation of cells did not lead to morphologic changes, and cells retained a similar speed of growth for several weeks after thawing.

**Table 1:** Donor information of all corneas used for culture of HCECs.

**Donor number**	**Age (years)**	**Days in Storage**	**Method**	**Success**
1	53	5	hydrodissection	no
2	53	5	hydrodissection	no
3	62	9	hydrodissection	no
4	72	7	hydrodissection	no
5	64	8	hydrodissection	no
6	64	5	hydrodissection	no
7	55	15	hydrodissection	no
8	55	15	hydrodissection	no
9	62	12	hydrodissection	no
10	66	7	hydrodissection	yes
11	55	7	hydrodissection	yes
12	63	6	hydrodissection	yes
13	17	14	hydrodissection	no
14	24	4	hydrodissection	no
Mean±SD	55±16	9±4		
15	38	7	forceps	no
16	38	7	forceps	yes
17	65	6	forceps	no
18	65	6	forceps	no
19	49	5	forceps	no
20	49	5	forceps	no
21	54	13	forceps	no
22	54	13	forceps	no
23	62	9	forceps	no
24	72	7	forceps	no
25	60	19	forceps	no
26	73	3	forceps	no
27	73	3	forceps	yes
28	46	10	forceps	no
29	62	12	forceps	no
30	66	7	forceps	yes
31	27	19	forceps	yes
32	27	19	forceps	yes
33	63	6	forceps	yes
34	17	14	forceps	no
35	24	4	forceps	yes
Mean±SD	52±17	9±5		

**Figure 3:**
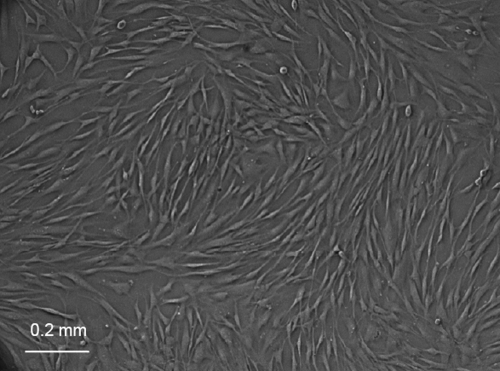
Human corneal stromal fibroblast cells after two weeks in culture before first passage. Cells exhibited a characteristic spindle-shaped appearance.

Stromal fibroblasts started growing out of corneal buttons after approximately one week in culture. They exhibited a typical, spindle-shaped appearance, initially reached confluence within one additional week in culture (Figure 3), and showed clear distinction from cultured HCECs.

### Reverse transcriptase polymerase chain reaction and sequencing of *COL8A2* cDNA

**Figure 4:**
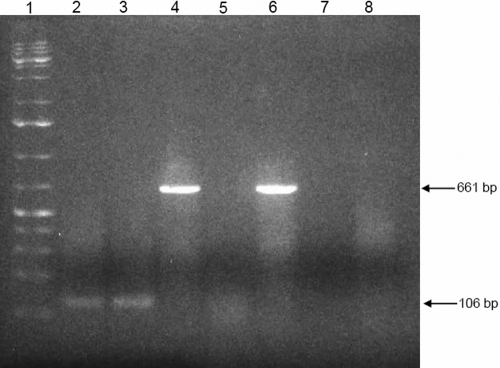
RT-PCR of total RNA of HCECs and human mononuclear cells for *COL8A2* and *β-actin*. Agarose gel shows in lane 1: 1 kb plus ladder, lane 2: *COL8A2* with HCECs, lane 3: *COL8A2* with HCECs, pretreated with DNase (control to rule out DNA contamination), lane 4: *β-actin* (housekeeping gene serving as positive control), lane 5: *COL8A2* with human mononuclear cells (negative control), lane 6; *β-actin* with human mononuclear cells (positive control), lane 7: no template (negative control to rule out contamination of reagents), lane 8: PCR with HiFi Taq of HCEC total RNA using *COL8A2* primers (negative control to rule out contamination of HCEC total RNA with genomic DNA).

A 106 base pair (bp) RT–PCR product of the size expected for *COL8A2* was obtained with cultured HCECs (Figure 4, lane 2). Pretreatment with DNase did not diminish the strength of the obtained band (Figure 4, lane 3). Sequence analysis of the obtained RT–PCR product showed 100% homology to the human *COL8A2* cDNA sequence (UCSC Genome Bioinformatics). RT–PCR of HCEC total RNA with commercially available *β-actin* primers yielded a band of the expected 661 bp size (Figure 4, lane 4). Human mononuclear cell total RNA was negative for *COL8A2* and positive for *β-actin* on RT–PCR analysis (Figure 4, lanes 5 and 6).

### Transfection

#### Lipid-/Liposome-mediated reagents

Transfection of pIRES-EGFP plasmid with Fugene and lipofectin each showed a rate of transfection of approximately 5%. For Fugene, optimal results were obtained with an incubation time of 60 min, 1 µg DNA, and 6 µl Fugene. With lipofectin, the most successful parameters were 2 µg DNA and 20 µl lipofectin with an incubation time of 6 h. The addition of PlusReagent to lipofectin did not significantly alter the rate of transfection. The rate of transfection with Fugene and lipofectin (with and without PlusReagent) was too small to yield a positive selection by the addition of neomycin. Different parameters led to a lower success of transfection. Transfection of pEGFP-N1 with GenCarrier2 showed the highest rate of transfection of all tested chemical reagents and was approximately 20%. For GeneCarrier2, the optimal rate of transfection was obtained with 3 µg DNA and 18 µg GenCarrier2 with an incubation time of 45 min (cell suspension). Cell toxicity of the reagents was low (~5%) and showed a concentration dependence.

#### Electroporation and cell-sorting

In electroporation experiments with the BTX method using pEGFP-N1 plasmid, the highest yield was obtained with a setting of three electric pulses and an interval of 100 ms, pulse length of 1 ms, and a voltage of 150 V. Transfection rate with these parameters was about 20%. With the second setting tested (as described above), success was considerably lower. Further evaluation was not performed.

**Figure 5:**
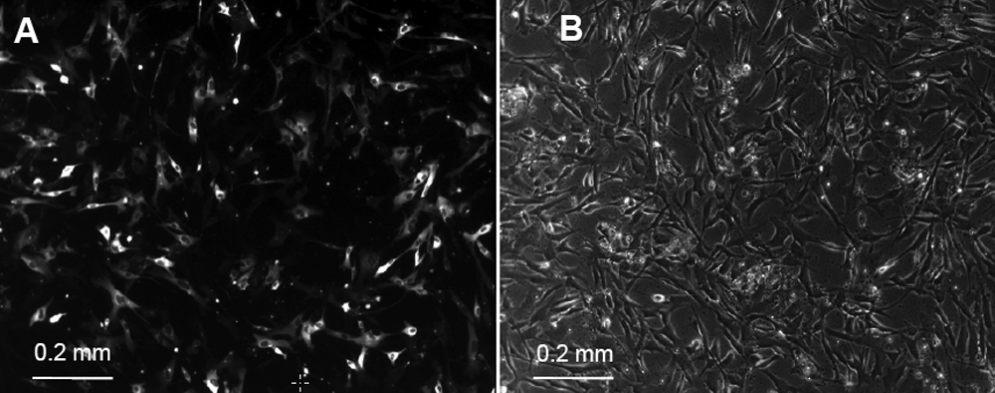
Human corneal endothelial cells on day 5 after transfection with Amaxa. Fluorescence microscopy (**A**) shows a transfection rate of ~30% compared with corresponding bright-field image (**B**).

With the Amaxa nucleofector using pmaxGFP, the rate of transfection varied from 20%–30% in the first two to four days after transfection, depending on the electrical program used as assessed by fluorescence microscopy. By day 5, rate of transfection was 30% for all of the five electrical settings, M-03, T-05, T-23, T-27, and U-11 (Figure 5). Electroporation with Amaxa was performed once for each program. Four additional replicates were performed using programs T-23 and T-27 only (see Methods). Variation in transfection efficiency was dependent on the cell count used for each electroporation procedure. Final cell count used for Amaxa ranged between 4×10^5^ and 6×10^5^ per reaction.

**Figure 6:**
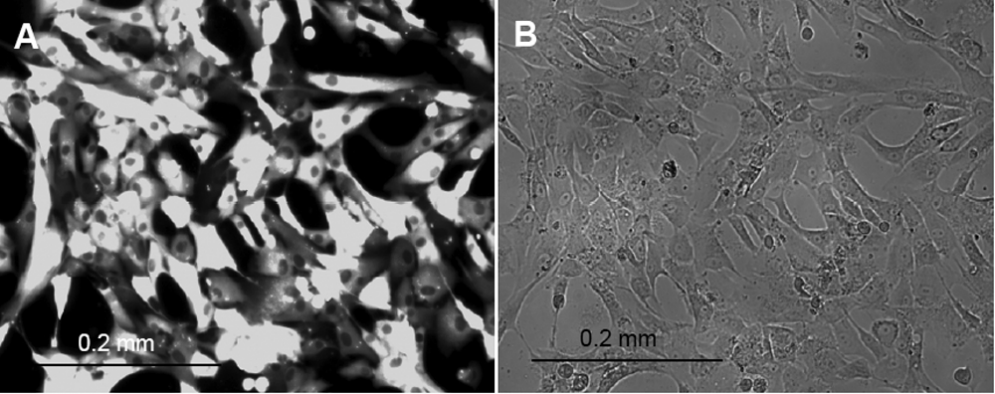
Human corneal endothelial cells transfected with Amaxa using pmaxGFP on day 3 after FACS. Fluorescence microscopy (**A**), exhibits an essentially pure population of transfected cells as compared with corresponding bright-field image (**B**).

BTX- and Amaxa-related cell death was approximately 10%. In cells transfected with the pEGFP-N1 plasmid (with neomycin resistance gene), we added neomycin after five days (as fluorescence increased after transfection for approximately five days). After 10 days, neomycin was removed from the medium. Removal of neomycin did not lead to sufficient recovery of the cells. Selection with FACS after electroporation with Amaxa was performed four times and led to a nearly homogeneous population of transfected cells (Figure 6). Gate settings were chosen to select a pure population of GFP-positive cells. These settings yielded a percentage of 22%±14% (mean±SD) GFP-positive cells.

## Discussion

In this work, methods of harvesting, genetically modifying, and enriching populations of HCECs were directly compared. Our main focus was to report optimal methods that are technically feasible and most efficient for research and potentially clinical uses. The challenges of establishing and maintaining primary HCEC cultures make such optimal methods highly relevant. Furthermore, HCEC lines are limited reagents, which make extensive analyses more difficult than for immortalized cell lines.

For harvesting HCECs, two different techniques were assessed. The separation of DM by hydrodissection yielded fewer viable cells than stripping with forceps. It seems plausible that the stretching of DM while the bleb is forming could damage the endothelial cells. In regard to clinical uses, especially in the context of Descemet membrane endothelial keratoplasty (DMEK), this observation could be of great importance [10] as hydrodissection as used in this study is also a convenient and reliable way of harvesting donor DM for possible use in DMEK (11). Further studies will be necessary to assess the risks of damaging HCECs by hydrodissection of DM before using this technique for clinical applications.

Previous studies of HCEC genetic modification have primarily focused on viral methods including adenovirus, adeno-associated virus, herpes simplex virus (HSV), human immunodeficiency virus (HIV), equine infectious anemia virus (EIAV), and prototypic foamy virus (PFV) [5,12-14] (Table 2). Viral transfection bears potential risks of inappropriate viral replication or induction of host immune responses [15]. Furthermore, use of retroviruses requires special safety precautions and bears the theoretical risk of insertional mutagenesis. Thus, non-viral techniques could be a safer and more practical approach. However, a drawback of non-viral techniques is generally lower transfection efficiencies and lower transgene expression. Additionally, because no integration in the host genome occurs, gene expression is typically transient. Further selection by use of resistance markers could be used to obtain stably expressing cells that have incorporated the transgene in their genome [16,17].

**Table 2:** Previous studies of human corneal endothelial cell genetic modification.

**Method**	**Rate of transfection**	**Reference**
**Viral vectors**		
adeno-associated virus, HSV	2%–5%	[12]
EIAV	30%	[5]
EIAV, adenovirus, HIV, PFV	43%–99%	[13]
adenovirus	up to 96%	[14]
**Lipid-/liposome-/peptide-mediated**		
Lipofectin	17%±2%	[18]
DMRIE-C	11%±1%	[18]
Effectene	9%±1%	[18]
Fugene	9%±1%	[18]
DAC-30	7%±1%	[18]
peptides (polyamidoamine)	2%	[19]
**Electroporation**		
GenePulser (BioRad)	area of clonal proliferation	[20]
Easyject Plus system (Eurogentec)	2.4%	[21]
GenePulser (BioRad)	large number of clones	[22]

In previously published studies, transfection with chemical reagents varied highly in efficiency depending on the reagents used [18,19] (Table 2). The most successful rate of transfection was obtained with lipofectin and was 17% as reported by Dannowski et al. [18]. Our transfection rates were lower using this reagent. The difference in the outcome might be due to our assessing the transfection rate by fluorescence microscopy and using a plasmid with a relatively low GFP expression level (pIRES-EGFP). Using GenCarrier2 with pEGFP-N1, our transfection rates were in the same range (approximately 20%) as in other studies using lipid-based reagents. Selection by antibiotics did not prove to be successful due to the relatively low rates of transfection in the liposome-/lipid-mediated transfection methods. One explanation for this may be that the transfection reagents increased the sensitivity of the cells to the antibiotic. This is unlikely as the antibiotic was added 48 h post-transfection, and the cells did not appear to have undue toxicity at this time point post-transfection.

Previous publications on electroporation methods reported a lower efficiency than in our work [20,21]. However, the previously described methods used different electroporation techniques. With both BTX and Amaxa, we received a higher efficiency than with the chemical reagents tested in this study. Our results also showed that toxicity was present but lower than 20% as has been reported in several previous studies on electroporation [19-21]· In our study, the rate of transfection using electroporation is efficient enough to select the transfected cells by flow cytometry to a nearly pure population.

Homogeneous, genetically modified HCECs could serve as a promising therapeutic tool. These modifications could include approaches to alter HCEC immunogenicity to prevent allograft rejection or enhance proliferative capacities to prevent cell loss after endothelial keratoplasty [23,24]. Some concern remains that the fibroblastic appearance after prolonged culturing could be due to de-differentiation. However, prior studies assessed that physiologic functions of cultured HCEC (such as the pump function of Na-K-ATPase or zonula occludens-1 expression) resemble those in vivo [9,25]. In addition, relatively early passages of cells could be used to minimize the effects of possible morphologic or functional changes before therapeutic use.

Early reports have described efforts to grow HCECs on artificial matrices and transfer these grafts as sheets into animal eyes or onto human donor corneas [25-27]. Another potentially useful approach could involve seeding genetically modified HCECs without a supporting material directly onto in vivo corneal stroma denuded of pathologic endothelium and/or DM [7,28,29]. Techniques to genetically modify and select HCECs as shown in this study could lead to improved success of future endothelial transplant procedures.

## References

[1] Adamis AP, Filatov V, Tripathi BJ, Tripathi RC (1993). Fuchs' endothelial dystrophy of the cornea.. Surv Ophthalmol.

[2] Joyce NC (2005). Cell cycle status in human corneal endothelium.. Exp Eye Res.

[3] Biswas S, Munier FL, Yardley J, Hart-Holden N, Perveen R, Cousin P, Sutphin JE, Noble B, Batterbury M, Kielty C, Hackett A, Bonshek R, Ridgway A, McLeod D, Sheffield VC, Stone EM, Schorderet DF, Black GC (2001). Missense mutations in COL8A2, the gene encoding the alpha2 chain of type VIII collagen, cause two forms of corneal endothelial dystrophy.. Hum Mol Genet.

[4] Gottsch JD, Sundin OH, Liu SH, Jun AS, Broman KW, Stark WJ, Vito EC, Narang AK, Thompson JM, Magovern M (2005). Inheritance of a novel COL8A2 mutation defines a distinct early-onset subtype of fuchs corneal dystrophy.. Invest Ophthalmol Vis Sci.

[5] Temin HM (1990). Safety considerations in somatic gene therapy of human disease with retrovirus vectors.. Hum Gene Ther.

[6] Zhu C, Joyce NC (2004). Proliferative response of corneal endothelial cells from young and older donors.. Invest Ophthalmol Vis Sci.

[7] Joyce NC (2003). Proliferative capacity of the corneal endothelium.. Prog Retin Eye Res.

[8] Suh LH, Zhang C, Chuck RS, Stark WJ, Naylor S, Binley K, Chakravarti S, Jun AS (2007). Cryopreservation and lentiviral-mediated genetic modification of human primary cultured corneal endothelial cells.. Invest Ophthalmol Vis Sci.

[9] Chen KH, Azar D, Joyce NC (2001). Transplantation of adult human corneal endothelium ex vivo: A morphologic study.. Cornea.

[10] Melles GR, Ong TS, Ververs B, van der Wees J (2006). Descemet membrane endothelial keratoplasty (DMEK).. Cornea.

[11] Sikder S, Lee J, Jun AS. Assessment of techniques to harvest donor tissue for Descemet’s membrane endothelial keratoplasty. ARVO Annual Meeting; 2008 April 27-May 1; Fort Lauderdale (FL).

[12] Hudde T, Rayner SA, De Alwis M, Thrasher AJ, Smith J, Coffin RS, George AJ, Larkin DF (2000). Adeno-associated and herpes simplex viruses as vectors for gene transfer to the corneal endothelium.. Cornea.

[13] Beutelspacher SC, Ardjomand N, Tan PH, Patton GS, Larkin DF, George AJ, McClure MO (2005). Comparison of HIV-1 and EIAV-based lentiviral vectors in corneal transduction.. Exp Eye Res.

[14] Schonthal AH, Hwang JJ, Stevenson D, Trousdale MD (1999). Expression and activity of cell cycle-regulatory proteins in normal and transformed corneal endothelial cells.. Exp Eye Res.

[15] Byrnes AP, Rusby JE, Wood MJ, Charlton HM (1995). Adenovirus gene transfer causes inflammation in the brain.. Neuroscience.

[16] Kodama K, Katayama Y, Shoji Y, Nakashima H (2006). The features and shortcomings for gene delivery of current non-viral carriers.. Curr Med Chem.

[17] Jo J, Tabata Y (2008). Non-viral gene transfection technologies for genetic engineering of stem cells.. Eur J Pharm Biopharm.

[18] Dannowski H, Bednarz J, Reszka R, Engelmann K, Pleyer U (2005). Lipid-mediated gene transfer of acidic fibroblast growth factor into human corneal endothelial cells.. Exp Eye Res.

[19] Hudde T, Rayner SA, Comer RM, Weber M, Isaacs JD, Waldmann H, Larkin DF, George AJ (1999). Activated polyamidoamine dendrimers, a non-viral vector for gene transfer to the corneal endothelium.. Gene Ther.

[20] Wilson SE, Lloyd SA, He YG, McCash CS (1993). Extended life of human corneal endothelial cells transfected with the SV40 large T antigen.. Invest Ophthalmol Vis Sci.

[21] Bednarz J, Teifel M, Friedl P, Engelmann K (2000). Immortalization of human corneal endothelial cells using electroporation protocol optimized for human corneal endothelial and human retinal pigment epithelial cells.. Acta Ophthalmol Scand.

[22] Wilson SE, Weng J, Blair S, He YG, Lloyd S (1995). Expression of E6/E7 or SV40 large T antigen-coding oncogenes in human corneal endothelial cells indicates regulated high-proliferative capacity.. Invest Ophthalmol Vis Sci.

[23] Beutelspacher SC, Pillai R, Watson MP, Tan PH, Tsang J, McClure MO, George AJ, Larkin DF (2006). Function of indoleamine 2,3-dioxygenase in corneal allograft rejection and prolongation of allograft survival by over-expression.. Eur J Immunol.

[24] Kikuchi M, Harris DL, Obara Y, Senoo T, Joyce NC (2004). p27kip1 antisense-induced proliferative activity of rat corneal endothelial cells.. Invest Ophthalmol Vis Sci.

[25] Sumide T, Nishida K, Yamato M, Ide T, Hayashida Y, Watanabe K, Yang J, Kohno C, Kikuchi A, Maeda N, Watanabe H, Okano T, Tano Y (2006). Functional human corneal endothelial cell sheets harvested from temperature-responsive culture surfaces.. FASEB J.

[26] Lai JY, Chen KH, Hsiue GH (2007). Tissue-engineered human corneal endothelial cell sheet transplantation in a rabbit model using functional biomaterials.. Transplantation.

[27] Ishino Y, Sano Y, Nakamura T, Connon CJ, Rigby H, Fullwood NJ, Kinoshita S (2004). Amniotic membrane as a carrier for cultivated human corneal endothelial cell transplantation.. Invest Ophthalmol Vis Sci.

[28] Bohnke M, Eggli P, Engelmann K (1999). Transplantation of cultured adult human or porcine corneal endothelial cells onto human recipients in vitro. part II: Evaluation in the scanning electron microscope.. Cornea.

[29] Amano S (2003). Transplantation of cultured human corneal endothelial cells.. Cornea.

